# Duplex Recombinase-Aided Amplification–Lateral Flow Dipstick (dRAA-LFD) Assay for New Zealand Green-Lipped Mussel Authentication

**DOI:** 10.3390/bios16030138

**Published:** 2026-02-27

**Authors:** Jirakrit Saetang, Maturada Saengthong, Soottawat Benjakul, Gururaj Moorthy

**Affiliations:** International Center of Excellence in Seafood Science and Innovation, Faculty of Agro-Industry, Prince of Songkla University, Hat Yai 90110, Thailand

**Keywords:** authenticity, seafoods, rapid test, isothermal amplification, bivalves

## Abstract

New Zealand green-lipped mussel (*Perna canaliculus*) is a premium seafood product that may be substituted with morphologically similar mussels after shucking and cooking, particularly Asian green mussel (*Perna viridis*). This study developed a rapid, on-site duplex recombinase-aided amplification–lateral flow dipstick (dRAA–LFD) assay to authenticate *P. canaliculus* and differentiate it from *P. viridis*. Species-specific primers were designed from mitochondrial COI alignment and combined in a dRAA reaction. Reaction conditions were optimized at 37–42 °C and 15–60 min. Specificity was assessed against 11 non-target seafood species, and sensitivity was evaluated using 2-fold serial dilutions. The assay was further validated using DNA from boiled (85 °C, 5–15 min), steamed (105 °C, 10–30 min), and fried (185 °C, 30–90 s) mussels, and 15 restaurant products labeled as New Zealand mussel dishes. Optimal performance was achieved at 40 °C for 30 min, with no cross-reactivity. The LFD detection limits were 0.05 ng/reaction for *P. viridis* and 0.2–0.1 ng/reaction for *P. canaliculus*. All cooked samples remained identifiable, and commercial testing classified 13/15 products as *P. canaliculus* and 2/15 as *P. viridis*. Overall, the dRAA–LFD assay enables rapid, equipment-light authentication of cooked mussel products for routine screening.

## 1. Introduction

New Zealand green-lipped mussel (*Perna canaliculus*) is the flagship species of the New Zealand aquaculture industry and contributes substantially to national seafood exports and regional economies [1]. Mussel farming now represents the cornerstone of New Zealand aquaculture, with New Zealand green-lipped mussel forming the bulk of production and export value, and industry projections aiming to increase aquaculture export earnings to NZ$3 billion by 2035 [1,2]. New Zealand green-lipped mussels are widely marketed as high-value frozen half-shell, ready-to-cook, and ready-to-eat products for export and premium foodservice markets, often commanding higher prices than generic mussel products in retail and restaurant channels [3]. Commercial positioning emphasizes provenance (“New Zealand Greenshell™”), sustainability credentials, and sensory quality, reinforcing their image as a premium seafood choice rather than a bulk commodity ingredient [2,3]. In such high-value contexts, economic incentives for species substitution and mislabeling are strong, particularly where cheaper mussel species can be presented as Greenshell™ mussels once shells are removed or when products are marinated, breaded, or incorporated into composite dishes [4,5,6]. Seafood fraud studies consistently show that substitution rates tend to increase along the value chain and in out-of-home catering, where visual inspection by consumers is limited [6,7,8,9]. Against this backdrop, robust analytical tools to authenticate species identity in Greenshell™ mussel food products are essential to protect consumers and support regulatory enforcement in international trade [10,11].

In contrast, the Asian green mussel (*Perna viridis*) is a widely cultured, relatively low-priced bivalve across tropical and subtropical coasts of Southeast Asia, including Thailand, Indonesia, the Philippines and neighbouring countries [12,13]. Green mussel farming is an important small- to medium-scale aquaculture activity that provides affordable animal protein and local employment, with production commonly based on simple rope or longline systems in coastal [14,15,16]. In Thailand, molluscan aquaculture, including *P. viridis*, accounts for a substantial share of coastal aquaculture volume and contributes significantly to domestic seafood supply [14,16]. Owing to its relatively low production costs and widespread availability, *P. viridis* is commonly used in street food, mass-market frozen products, and catering, where it is sold fresh, blanched, or as shucked meat [5]. When mussels are de-shelled, cooked, or incorporated into seasoned dishes, morphological differences between *P. canaliculus* and *P. viridis* are generally unreliable for species authentication. Although subtle variations in color tone or tissue appearance may occasionally be observed, such characteristics are not consistent across samples or processing conditions and cannot be used as definitive criteria for species identification. This scenario reflects a broader pattern seen in seafood markets, where lower-value species are substituted for higher-value taxa in ready-to-eat or processed foods, leading to economic fraud and potential non-compliance with labelling regulations [8,10,17]. Recent molecular surveillance studies demonstrate that seafood mislabeling remains a widespread problem. In Thailand, DNA barcoding analyses of imported seafood products revealed mislabeling frequencies of approximately 20–30%, particularly among high-value and processed items [8]. For mussel products, targeted authentication of frozen Chilean blue mussels showed that only 50% of commercial brands contained 100% authentic *Mytilus chilensis*, while the remaining brands contained other mytilid species at proportions ranging from 12.5% to 50% [18]. Although several studies have demonstrated this kind of mislabeling, dedicated methods to distinguish *P. canaliculus* from *P. viridis* directly in food matrices remain limited, underscoring the need for practical species authentication assays tailored to mussel food products in regional and international supply chains.

Molecular authentication has emerged as the most reliable approach for seafood species identification, especially when traditional morphological traits are lost during shucking, cooking, mincing, or other processing steps typical of ready-to-cook and ready-to-eat foods. DNA barcoding based on mitochondrial cytochrome c oxidase subunit I (COI) and related markers provides a standardized framework for animal species identification and has been widely adopted in fish and shellfish supply chains [10,19,20]. Large-scale barcoding surveys have documented seafood mislabeling in retail and foodservice settings across Europe, North America and Asia, demonstrating both the scale of the problem and the utility of molecular tools for enforcement [21,22,23,24]. For mussels, PCR-based identification methods using both nuclear and mitochondrial markers have been widely established for fresh and processed food products, including a two-step PCR-restriction fragment length polymorphism (RFLP) approach based on 18S rDNA, ITS1 and the adhesive foot protein gene or PCR-RFLP protocols using the adhesive foot protein gene and COI [25,26]. Building on these foundations, a variety of rapid molecular methods, including real-time PCR, multiplex barcoding assays, and high-resolution melt analysis, have been implemented for bivalve authentication and mixed-species ingredient testing in food matrices [10,27]. Nonetheless, many of these platforms remain laboratory-based, require thermocyclers, real-time PCR instruments, or sequencing facilities, and may be too resource-intensive for routine on-site verification in processing plants, border inspection posts, or local markets, highlighting the need for simpler, faster, and field-deployable molecular tests [28,29,30].

Recombinase-aided amplification (RAA) represents a newer generation of isothermal nucleic acid amplification that offers several advantages over conventional PCR for food authentication, particularly when combined with lateral flow dipstick (LFD) read-out. When coupled with LFD strips, RAA products can be visualized by eye through labelled probes or primers, producing qualitative band-type results analogous to immunochromatographic tests, with minimal equipment and operator training [31,32]. RAA-LFD assays have been successfully applied for the rapid detection of viral and bacterial pathogens and for quarantine inspection and food safety monitoring, demonstrating high analytical sensitivity, robust performance in crude extracts, and suitability for use outside conventional laboratories [31,32,33]. Parallel developments in recombinase polymerase amplification (RPA) coupled with LFD have shown that isothermal amplification can effectively authenticate food species, underscoring the broader potential of isothermal–LFD platforms for food fraud control [30,34]. Adapting these principles, a duplex RAA-LFD (dRAA-LFD) format targeting species-diagnostic loci provides a promising strategy to simultaneously amplify and visually discriminate *P. canaliculus* and *P. viridis* in shucked, cooked, or frozen mussel products.

To address this gap, the present study aimed to develop a duplex recombinase-aided amplification–lateral flow dipstick (dRAA-LFD) assay targeting species-diagnostic loci for the detection and differentiation of *P. canaliculus* and *P. viridis* in mussel food products. Specifically, the objectives were to design and optimize species-specific dRAA-LFD primers and evaluate the analytical sensitivity, specificity, and robustness of the assay using purified DNA and thermally processed or frozen mussel matrices. Moreover, this work assessed its applicability for the routine screening of commercial New Zealand green-lipped mussel products as a rapid tool to support species authentication and fraud control.

## 2. Materials and Methods

### 2.1. Sample Preparation

Fresh and frozen specimens from two mussel species, including New Zealand green-lipped mussel (*P. canaliculus*) and Asian green mussel (*P. viridis*), were sourced from a local fresh market in Songkhla, Thailand, as well as from online retailers. All samples were transported to the laboratory in a frozen state. Species identity was verified on the basis of shell morphology using the description guidelines provided by the Sealifebase database (www.sealifebase.org, version 04/2025; accessed on 5 April 2025). The edible tissue was carefully removed from the shells, transferred into sterile plastic bags, and stored at −80 °C until further use.

To simulate different culinary and industrial conditions, the edible portions of each mussel species were subjected to three thermal processing treatments: boiling, steaming and frying. For boiling, samples were heated in a temperature-controlled water bath (Esstell, Namyangju-SI, Republic of Korea) at 85 °C for 5, 10 or 15 min. For steaming, samples were cooked in a steaming pot at 105 °C for 10, 20 or 30 min. For frying, samples were immersed in palm oil at 185 °C for 30, 60 or 90 s. All thermally treated mussel samples were kept at 4 °C and analysed within two days. For each processing condition, two independent samples per species were prepared to evaluate the specificity and repeatability of the developed assay.

### 2.2. DNA Extraction

Genomic DNA was extracted from raw and thermally processed mussel samples using the QIAamp DNA Mini Kit (Qiagen, Hilden, Germany) according to the manufacturer’s instructions, with minor adaptations. Briefly, approximately 30 mg of minced tissue was subjected to Proteinase K digestion, followed by column-based purification. Genomic DNA was eluted using the supplied elution buffer. DNA concentration and purity were assessed using a NanoDrop™ Lite spectrophotometer (Thermo Fisher, Waltham, MA, USA), and the extracted DNA was stored at −20 °C until analysis.

### 2.3. Designing of RAA Primers

RAA primers for the amplification of mussel DNA were designed based on the mitochondrial COI gene sequences of *P. viridis* and *P. canaliculus*, which were retrieved from the GenBank database with accession numbers 13435563 and 63651044, respectively. All sequences were aligned by using Clustal Omega via EMBL-EBI web server (https://www.ebi.ac.uk/jdispatcher/msa/clustalo; accessed on 24 April 2025) to identify the unique region. In accordance with general RAA design principles, primers were selected with a length of approximately 30–35 nucleotides, avoiding palindromic motifs, and targeting amplicons in the range of 100–200 bp. Candidate primers were required to have a GC content of 40–50% and to be free from significant self-dimer formation or secondary structures. Primer properties were evaluated using the OligoAnalyzer tool provided by Integrated DNA Technologies (Coralville, IA, USA). In total, two RAA primer pairs were generated, each directed to a specific region of the COI gene (Table 1). Species-specific primers were designed within conserved regions of the target mussel species using Primer3Plus version 3.3.0 (https://www.primer3plus.com; accessed on 25 April 2025), and their specificity was verified by the NCBI BLASTn web server against the nt database (accessed on 25 April 2025).

### 2.4. RAA-LFD Assay

RAA reactions were set up in a final volume of 25 μL using a commercial RAA nucleic acid amplification kit (Qitian Gene Biotechnology, Wuxi, China), following the manufacturer’s protocol. Each reaction contained 12.5 μL of buffer V, 0.2 μM of forward primer, 0.2 μM of reverse primer, and 7 μL of nuclease-free water. This mixture was then added to the tube containing the lyophilized enzyme pellet and briefly vortexed to dissolve. Subsequently, 50 ng of DNA template and 2.5 μL of magnesium acetate (280 mM) were pipetted onto the inner side of the tube cap, after which the tubes were closed, vortexed, and gently spun down to initiate the reaction. Incubation was performed in a mini dry bath incubator. The resulting RAA amplicons were separated on 2% agarose gels and visualized after staining with ethidium bromide. The amplified products were confirmed by Sanger sequencing.

The integrated nucleic acid–based strip detection system was configured according to the following concept. Species-specific forward and reverse primers, each carrying distinct labels, were incorporated into the RAA reactions (Table 1). The resulting amplicons contain different combinations of tags that are designed to bind specifically to the two test lines on the DNA strip. For the New Zealand green-lipped mussel, the RAA product carries both CY5 and FAM labels, whereas the amplicon from the Asian green mussel is labeled with biotin and FAM. Biotin and CY5 selectively interact with anti-biotin and anti-CY5 antibodies immobilized at the first and second test lines, respectively. FAM serves as the fluorescent hapten that binds exclusively to the anti-FAM rabbit antibody conjugated to nanogold particles present in the reaction buffer. The control line on the strip, coated with anti-rabbit antibody, functions as an internal control to verify proper flow and conjugate performance.

The Customized Test Line Hybridetect DNA strip kit (Milenia Biotec, Giessen, Germany) was employed to detect the duplex RAA amplicons. For each test, 2.5 µL of the dRAA product was mixed with 100 µL of HybriDetect assay buffer (Milenia Biotec, Giessen, Germany), and a DNA strip was then inserted into the mixture. After incubating for 5 min, the appearance of the test (T) lines and the control (C) line was observed for result interpretation. In samples where the Asian green mussel is present, only the first test line will be positive. In contrast, the second test line will be positive when DNA from the New Zealand green-lipped mussel exists. Surplus nanogold conjugates are captured at the control line, which appears as a positive band confirming that the lateral flow device has functioned correctly.

### 2.5. Specificity and Sensitivity Tests

The analytical specificity of the developed duplex RAA–LFD assay was evaluated using genomic DNA from the two target mussel species together with a panel of non-target molluscs and crustaceans that are commonly present in mixed seafood products. The non-target panel comprised *Meretrix meretrix* (MM, hard clam), *Tegillarca granosa* (TG, blood cockle), *Paratapes undulata* (PU, undulated surf clam), *Trisidos semitorta* (TS, semi-twisted Ark), *Portunus pelagicus* (PP, blue swimming crab), *Portunus sanguinolentus* (PS, three-spot swimming crab), *Charybdis (Charybdis) feriata* (CF, crucifix crab), *Scylla serrata* (SS, mud crab), *Penaeus vannamei* (PS, Pacific whiteleg shrimp), *Penaeus monodon* (PM, giant freshwater prawn), and *Penaeus (Fenneropenaeus) merguiensis* (FM, banana shrimp). Genomic DNA from each species was subjected to the RAA–LFD assay under the same conditions used for the target mussels. RAA products were examined on 2% agarose gels prior to LFD analysis to verify successful amplification of the expected target fragments.

Sensitivity was evaluated by using both agarose gel electrophoresis and LFD for Asian green mussel and New Zealand green-lipped mussel. For each reaction type, the starting DNA input was 50 ng per reaction, followed by fifteen successive 2-fold serial dilutions down to 0.003 ng per reaction. The resulting amplicons from RAA were examined by agarose gel electrophoresis, with gels stained with ethidium bromide (1 µg/mL) and visualized under fluorescence.

## 3. Results

### 3.1. COI Sequence Alignment and Duplex RAA-LFD Optimization

**Figure 1:**
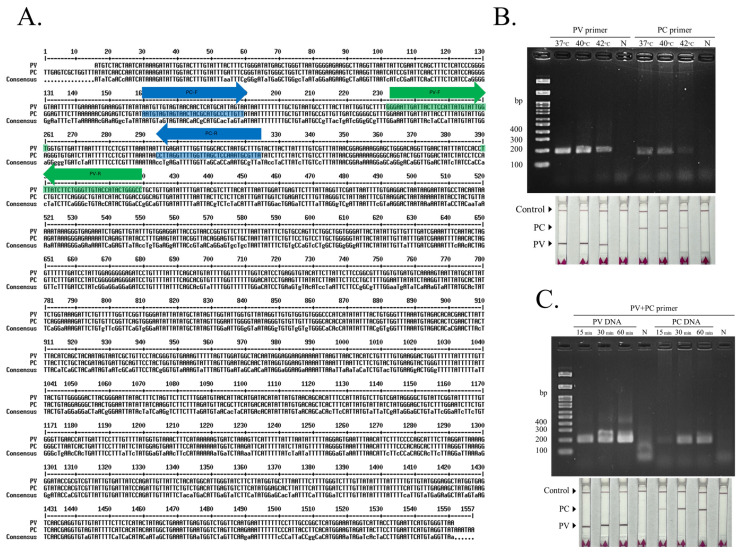
COI marker selection and optimization of the duplex RAA–LFD assay for discriminating Asian green mussel and New Zealand green-lipped mussel. (**A**) Clustal Omega alignment of mitochondrial COI sequences showing the primer-binding regions used to design species-specific primer sets (PV-F/PV-R and PC-F/PC-R) within the aligned COI fragment. (**B**) Optimization of RAA reaction temperature (37, 40, and 42 °C) using singleplex PV or PC primer sets. (**C**) Optimization of RAA reaction time (15, 30, and 60 min) using duplex primer reactions (PV + PC) with either PV or PC genomic DNA as template.

Clustal Omega alignment of the mitochondrial COI gene sequences of *P. viridis* and *P. canaliculus* (GenBank accessions 13435563 and 63651044) showed high sequence similarity (>75% identity) across the aligned region (Figure 1A). Despite the overall conservation, several species-diagnostic nucleotide polymorphisms were observed and used for species-specific primer-binding sites. To optimize the RAA reaction, singleplex RAA reactions were first evaluated at 37, 40, and 42 °C using the *P. viridis* (PV) primer set and the *P. canaliculus* (PC) primer set. The expected amplification products were detectable across the tested temperature range for both primer sets, as confirmed by agarose gel electrophoresis and the DNA strip system (Figure 1B). However, 40 °C produced the most intense and consistent bands on agarose gels and the clear test-line signals on the strips for both PV and PC assays, and no amplification signal was observed in the negative controls. Using the selected temperature condition, the reaction time was further optimized by incubating duplex primer reactions (PV + PC) for 15, 30, and 60 min with either PV DNA or PC DNA templates. Detectable amplification was observed as early as 15 min, and increased markedly at 30 min (Figure 1C). Extending incubation to 60 min did not provide a substantial improvement over 30 min in terms of interpretability. Therefore, 40 °C for 30 min was selected as the optimal condition for subsequent dRAA-LFD experiments.

### 3.2. Specificity and Sensitivity Evaluation of the dRAA-LFD System

**Figure 2:**
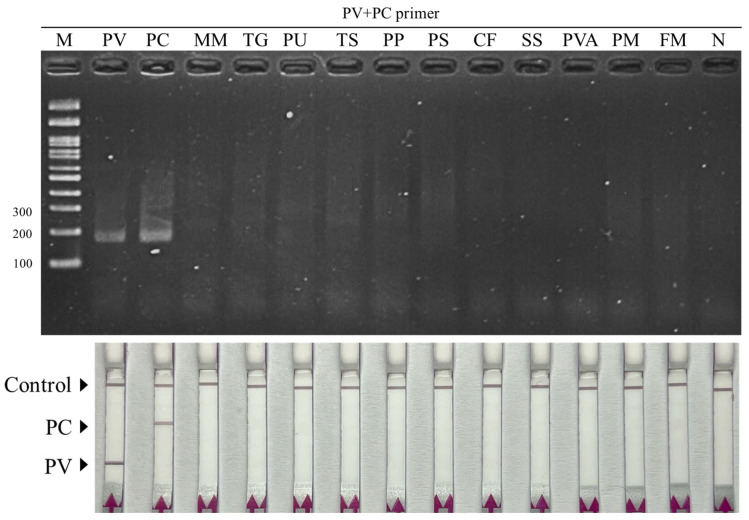
Specificity test of the duplex RAA–LFD assay. Representative 2% agarose gel electrophoresis (**top**) and corresponding LFD readout (**bottom**) obtained from target mussel DNA (*P. viridis* and *P. canaliculus*) and non-target seafood species. MM: *Meretrix meretrix* (hard clam), TG: *Tegillarca granosa* (blood cockle), PU: *Paratapes undulata* (undulated surf clam), TS: *Trisidos semitorta* (semi-twisted ark), PP: *Portunus pelagicus* (blue swimming crab), PS: *Portunus sanguinolentus* (three-spot swimming crab), CF: *Charybdis (Charybdis) feriata* (crucifix crab), SS: *Scylla serrata* (mud crab), PVA: *Penaeus vannamei* (Pacific whiteleg shrimp), PM: *Penaeus monodon* (giant tiger prawn), FM: *Penaeus (Fenneropenaeus) merguiensis* (banana shrimp).

The analytical specificity of the developed duplex RAA–LFD assay was assessed using genomic DNA from the two target mussel species (*P. viridis* and *P. canaliculus*) together with a panel of non-target molluscs and crustaceans commonly encountered in mixed seafood products. As shown in Figure 2 (upper panel), amplification was observed only when the target templates were used in the dRAA reaction, with clear RAA products detected on agarose gel, while no detectable amplification was observed for any non-target species. Consistently, LFD readout showed species-specific signals since samples containing Asian green mussel generated a positive band at the first test line (T1), whereas samples containing New Zealand green-lipped mussel produced a positive band at the second test line (T2) (Figure 2 lower panel). In contrast, all non-target species yielded only the control line, indicating the absence of cross-reactivity under the same assay conditions.

**Figure 3:**
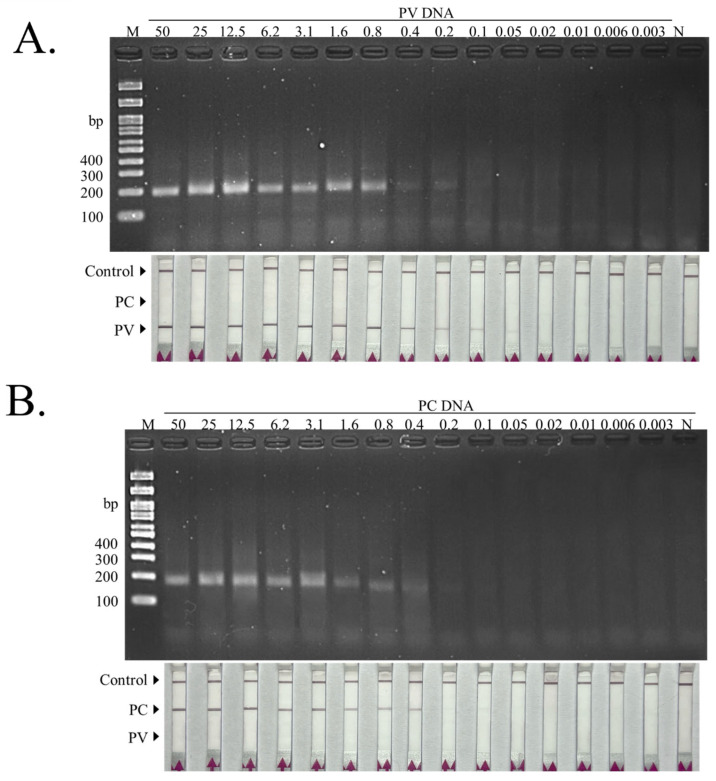
Analytical sensitivity of the dRAA–LFD assay. (**A**) *P. viridis* and (**B**) *P. canaliculus* genomic DNA were 2-fold serially diluted (50 to 0.003 ng/reaction) and tested by duplex RAA–LFD. Upper panels show representative 2% agarose gels of RAA products; lower panels show the corresponding dipstick readouts.

In addition, the analytical sensitivity of the duplex RAA–LFD assay was determined for Asian green mussel (*P. viridis*) (Figure 3A) and New Zealand green-lipped mussel (*P. canaliculus*) (Figure 3B) using a 2-fold serial dilution series of genomic DNA starting from 50 ng/reaction to 0.003 ng/reaction. For *P. viridis* (Figure 3A), the expected RAA band on agarose gel was observable from high-template inputs down to approximately 0.2 ng/reaction. The dipstick readout (T1 line) remained detectable across the dilution series, with a faint but visible test band observed down to 0.05 ng/reaction. At lower DNA inputs, the PV-specific test line was not observed, whereas the control line remained present. Similarly, for *P. canaliculus* (Figure 3B), the expected RAA product band was detected down to approximately 0.2 ng/reaction, whereas lower template inputs yielded no detectable band. The dipstick readout (T2 line) was detectable at high template concentrations, and a very faint test band was still observable at approximately 0.2–0.1 ng/reaction. Below this range, no PC-specific test line was visible, while the control line was consistently present across all reactions.

### 3.3. The dRAA-LFD Test of Cooked Samples

**Figure 4:**
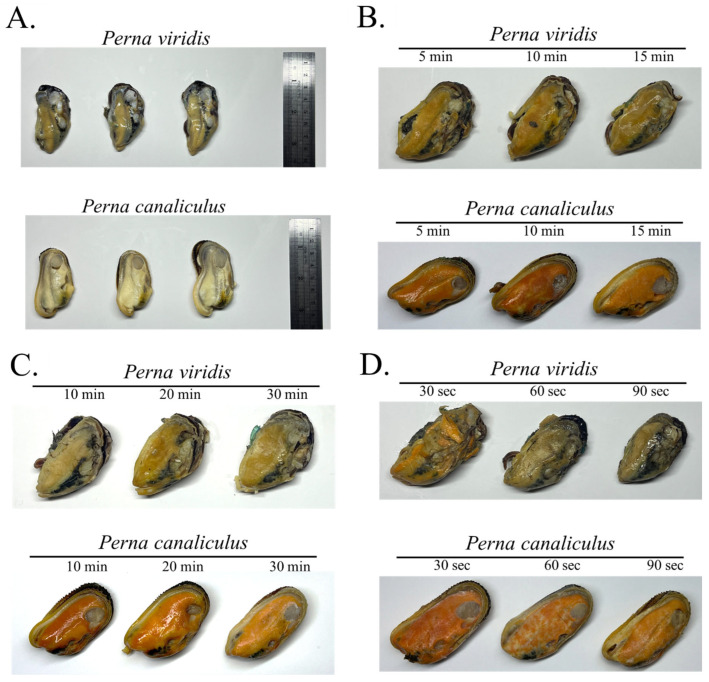
Macroscopic appearance of mussel samples before and after cooking. Representative images of (**A**) fresh shucked mussels, (**B**) boiled samples (85 °C; 5, 10, and 15 min), (**C**) steamed samples (105 °C; 10, 20, and 30 min), and (**D**) fried samples (185 °C; 30, 60, and 90 s).

**Figure 5:**
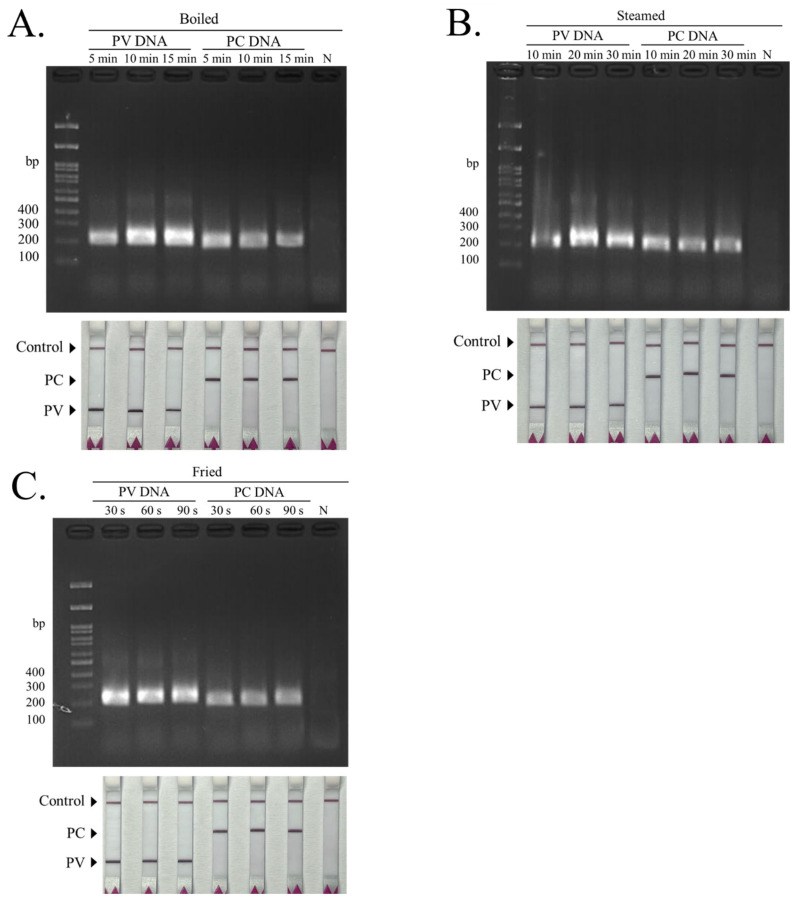
dRAA–LFD performance using DNA extracted from cooked mussel samples. Representative 2% agarose gels (**top**) and corresponding LFD readouts (**bottom**) for DNA from (**A**) boiled mussels (85 °C; 5–15 min), (**B**) steamed mussels (105 °C; 10–30 min), and (**C**) fried mussels (185 °C; 30–90 s), showing species-specific test-line patterns with a valid control line.

Representative images of shucked mussel tissues are shown for (Figure 4A) fresh shucked samples, (Figure 4B) boiled samples (85 °C; 5, 10, 15 min), (Figure 4C) steamed samples (105 °C; 10, 20, 30 min), and (Figure 4D) fried samples (185 °C; 30, 60, 90 s). Overall, thermal processing resulted in noticeable changes in tissue color and texture. Although macroscopic differences were observable among the samples and across processing conditions, these variations were neither distinct nor consistently reproducible to allow reliable discrimination of the two mussel species. However, the application of the dRAA–LFD system to purified DNA extracted from these cooked samples enabled correct identification of each species (Figure 5). Notably, successful amplification was achieved from samples steamed for up to 30 min or boiled for up to 15 min (Figure 5A,B), and high amplification efficiency was still observed for samples processed at temperatures as high as 185 °C (Figure 5C). For each processing condition, RAA products were detectable by agarose gel electrophoresis, and corresponding LFD readouts showed the presence of the control line with species-associated test-line patterns. Collectively, these findings demonstrate that the dRAA–LFD platform is suitable for seafood authentication across different cooking methods and treatment durations.

### 3.4. Application of the dRAA-LFD Method to Commercial Products

**Figure 6:**
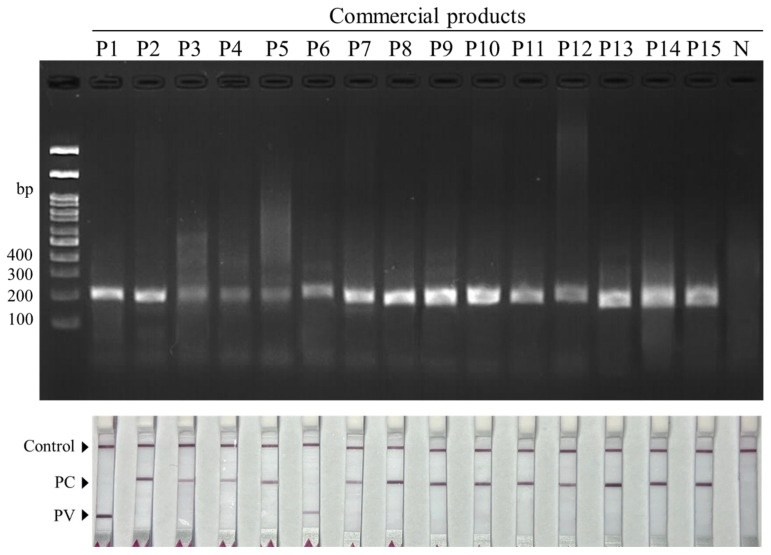
Application of the dRAA–LFD assay to commercial food products claiming New Zealand mussels. Representative 2% agarose gel electrophoresis of dRAA products (**top**) and corresponding LFD readouts (**bottom**) obtained from DNA extracted from 15 cooked restaurant products (P1–P15), showing species assignment based on the presence of *P. canaliculus*- or *P. viridis*-specific test lines together with the control line.

To evaluate field applicability, the developed dRAA–LFD assay was applied to 15 cooked mussel dishes (P1–P15) purchased from restaurants and marketed as using New Zealand green-lipped mussel. Purified DNA extracted from each product was subjected to dRAA amplification and read out by agarose gel electrophoresis and the duplex LFD format. As shown in the gel and dipstick results (Figure 6), amplification products were obtained from all commercial samples. The LFD readout identified 13/15 samples (86.7%) as *P. canaliculus* (T2 positive), consistent with the label claim. In contrast, two samples (P1 and P6) produced a T1-positive pattern, indicating *P. viridis* rather than *P. canaliculus* (Figure 6, lower panel and Table 2). Overall, the commercial-sample screening demonstrated that the dRAA–LFD assay generated interpretable results from ready-to-eat restaurant products and enabled species assignment in these processed matrices.

## 4. Discussion

Seafood mislabeling remains a persistent authenticity challenge, particularly for bivalve products that are frequently traded as shucked or further-processed tissues where diagnostic morphological traits are removed. Large-scale syntheses indicate that species substitution is one of the most common forms of seafood fraud globally, although product-level rates vary widely and data for invertebrates are still comparatively limited [35]. In the present study, visual inspection of New Zealand green-lipped mussels and Asian green mussels across multiple processing conditions showed that, while subtle macroscopic differences could sometimes be noticed, these variations were not sufficiently distinct or consistent to support reliable species discrimination. This observation aligns with prior work emphasizing that post-processing changes, including heat-induced color/texture shifts, tissue contraction, and loss of shell-associated cues, can erase or homogenize appearance-based identifiers, which increase the need for DNA-based confirmation at control points along the supply chain [36,37]. For assay development, reference specimens of New Zealand green-lipped mussel were selected from commercially packaged products with declared species names from reputable brands, whereas Asian green mussels were obtained from local markets and identified based on established morphological characteristics. Although this strategy was considered appropriate for method optimization, the incorporation of complementary molecular verification, such as COI DNA barcoding, would further enhance the robustness of reference material validation and may be implemented in future studies. Our results, therefore, reinforce a practical premise for market surveillance: once mussels are sold as cooked, seasoned, or ready-to-eat dishes, morphological screening is inherently unreliable and should be replaced or at least corroborated with molecular testing, especially in restaurant settings where labeling information is typically communicated only via menus or receipts rather than traceable packaging documentation [35,38,39].

The COI gene was selected as the primary marker because it is the cornerstone of animal DNA barcoding and supports broad comparability with curated reference libraries. Hebert et al. (2003) proposed COI as a standardized barcode for animal identification, demonstrating strong discriminatory capacity when robust reference datasets are available [40,41]. In seafood authentication, COI has been repeatedly adopted as an effective locus for differentiating closely related commercial taxa, including cephalopods and bivalves, particularly when assays are designed to exploit species-informative single-nucleotide variants within otherwise conserved regions [29]. Harris et al. (2016) used COI-based analysis to assess seafood mislabeling and reported that roughly 19% of the tested items were misrepresented, with particularly frequent issues observed among crustacean and bivalve products [42]. In addition, recent work has demonstrated the use of the mitochondrial COI gene as an authentication marker by developing an RAA–CRISPR/Cas12a assay for Pacific oyster (*Crassostrea gigas*), enabling rapid, highly specific detection across processed products and identifying 6.67% mislabeling in commercial samples [43]. In COI alignment, the two target mussel species shared high overall conservation while retaining multiple diagnostic polymorphisms within the primer/probe-binding windows, enabling the design of species-selective oligonucleotides that minimize cross-reactivity. At the same time, it is important to recognize limitations reported for certain mussel groups: mitochondrial markers can be complicated by introgression and atypical inheritance in *Mytilus* spp., which has prompted caution in marker choice and reference interpretation in some market-control contexts [4]. Although *Perna* spp. generally present clearer mitochondrial differentiation than *Mytilus* complexes, this literature underscores the need for careful reference curation, periodic in silico re-validation against expanding databases, and confirmatory sequencing for unexpected results [4,44].

From an analytical-performance standpoint, the dRAA–LFD platform offers a practical balance between specificity, operational simplicity, and turnaround time. RAA-based systems can be completed under mild isothermal conditions with minimal equipment, and coupling to LFD enables instrument-free interpretation at the point of need [26,45]. The method requires only a simple constant-temperature heating device (e.g., heat block or portable incubator), basic pipettes, and lateral flow dipsticks, without the need for a thermal cycler or gel electrophoresis equipment. These features substantially reduce infrastructure costs and technical complexity compared with conventional PCR-based authentication workflows [31,46,47]. From a cost perspective, the major consumables are the isothermal amplification reagents, primers, and lateral flow strips, which are generally less expensive than PCR master mixes, together with electrophoresis reagents and associated consumables. Moreover, the elimination of post-amplification gel analysis reduces both reagent use and labor time. As a result, the overall per-test cost is expected to be comparatively low, making the assay accessible for routine screening rather than only confirmatory laboratory testing.

Although PC and PV amplicons are similar in size and therefore indistinguishable by agarose gel electrophoresis, the duplex RAA–LFD system differentiates targets through species-specific primer labels rather than fragment length. The PC amplicon carries CY5-FAM labels, whereas the PV amplicon carries biotin–FAM labels, allowing capture on separate test lines of the LFD strip. Thus, if both species are present in the same reaction, two test lines are theoretically expected. During optimization, amplification remained robust from 37 to 42 °C, with 40 °C producing the most consistent gel bands and clearest lateral flow signals; a 30 min incubation gave improved interpretability over 15 min without meaningful gains at 60 min. Importantly, the duplex format was engineered to generate distinct test-line patterns on a single strip: PV-positive samples produced the T1 line, whereas PC-positive samples produced the T2 line. Analytical specificity testing against a panel of non-target molluscs and crustaceans yielded no detectable cross-reactivity. This specificity performance is comparable in principle to other COI-targeted isothermal assays reported for bivalves, such as the COI-based LAMP assay for multiple mussel species [5], which also relied on species-specific primer binding and showed clean discrimination among targeted taxa on gels and by colorimetric readout. Likewise, a COI-guided RAA–CRISPR/Cas12a assay for Pacific oyster authentication reported no cross-amplification with closely related oyster species under optimized conditions [43]. Although the non-target panel focused on bivalves and crustaceans commonly present in Thai seafood products, inclusion of additional closely related mytilids and *Perna* species would further enhance specificity assessment and should be explored in future studies. Collectively, these comparisons support that COI remains a robust marker for designing high-specificity assays, while our duplex LFD architecture adds practical value by enabling two-species discrimination in a single reaction and a single visual strip.

When benchmarked against existing approaches, the present assay produced detectable LFD signals down to 0.05 ng DNA per reaction for *P. viridis*, whereas *P. canaliculus* yielded faint but observable bands around 0.2–0.1 ng per reaction. These limits align with those reported for several DNA-based assays. As an example, a validated multi-locus PCR-HRM strategy for *Mytilus* spp. reported locus-dependent detection thresholds at 5–8 ng/µL DNA and highlighted matrix-specific constraints for some canned products [37]. Moreover, another study employed the RPA combined with a dipstick for the authentication of common octopus, which showed a limit of detection of 0.5 ng [29]. However, the developed assay remains less sensitive than CRISPR-enhanced recombinase systems; for example, an RAA–Cas12a assay targeting COI for oyster authentication reported detection down to the fg/reaction range alongside strong species exclusivity [43]. Practically, these detection limits remain suitable for most routine authenticity testing where DNA yields from bivalve tissues are typically above the sub-ng/reaction range, and the duplex format reduces test burden compared with running separate singleplex assays.

Beyond analytical metrics, practical deployment depends on operational simplicity, tolerance to processing-induced DNA degradation, and interpretability by non-specialists. Thermal processing can fragment DNA, alter extraction yields, and introduce inhibitors (lipids, salts, spices) as demonstrated by several studies. For example, Haunshi et al. (2009) reported that an 835 bp fragment could not be amplified from DNA extracted from meat treated at 121 °C for 20 min (15 psi) [48]. Similarly, Arslan et al. (2006) demonstrated that even comparatively shorter PCR products (≈271 bp) failed to amplify when DNA was extracted from beef fried at >190 °C for 80 min [49]. Therefore, performance of the assay must be demonstrated using representative cooked matrices rather than purified reference tissue alone [50,51]. In the present study, the duplex RAA–LFD assay remained applicable to DNA extracted from mussel samples exposed to common culinary treatments (boiling, steaming, and frying) across multiple time/temperature conditions. This aligns with broader evidence that well-designed short-amplicon assays can retain functionality after heat treatment; for example, standardized real-time PCR plate assays have shown strong robustness after controlled heat processing (95 °C for 20 min) while maintaining sensitivity in complex food matrices [52]. O et al. (2009) systematically showed that cooking substantially reduced nuclear DNA yield and degraded DNA quality, consistent with heat-driven fragmentation [53]. They further reported that large PCR targets (>800 bp) were only recoverable from raw or lower-temperature cooked meat, whereas small amplicons (<200 bp) could still be amplified across all tested core temperatures (75–100 °C), supporting the design principle that cooked-sample authentication assays should prioritize short amplicons to maintain amplification success when DNA is fragmented and nuclear DNA abundance is limited. Collectively, these comparisons suggest that duplex RAA–LFD provides a pragmatic balance, high species specificity with instrument-light readout and demonstrated applicability to cooked products, positioning it as a practical screening tool that complements (rather than replaces) higher-infrastructure approaches (e.g., real-time PCR/HRM or CRISPR-enhanced assays) when confirmatory sensitivity or multiplexing is required.

## 5. Conclusions

This work provides a practical dRAA–LFD workflow to discriminate *P. canaliculus* and *P. viridis* using a COI-based design and a simple visual readout. The results highlight that macroscopic inspection is unreliable after common culinary processing, whereas short-amplicon, isothermal amplification remains workable from cooked matrices across multiple time/temperature conditions. The duplex strip format enables single-tube, species-resolved interpretation without specialized instrumentation, supporting rapid screening in settings where conventional PCR-based confirmation is impractical. The detection limits observed for the LFD endpoint also indicate a trade-off between instrument-free convenience and ultra-high analytical sensitivity achievable by fluorescence/CRISPR platforms. While the current work represents a laboratory-based validation, the simplicity of the workflow, minimal equipment requirements, and visual readout suggest potential applicability as a preliminary screening tool in seafood processing facilities, markets, and inspection settings. Future studies incorporating a broader range of target and non-target species, mixed-species samples, and multi-site validation will be valuable to further define the scope and operational performance of the assay. Overall, the assay can serve as a frontline screening tool to support routine authenticity checks, with discrepant or borderline results ideally verified by sequencing or laboratory-based methods in future surveillance programs.

## Figures and Tables

**Table 1:** Primers selected for the detection and differentiation of New Zealand green-lipped mussel (PC) and Asian green mussel (PV).

Primer		Sequence (5′-3′)	Amplicon Size (bp)
PC	F	5′CY5 NHS Ester-AATGTAGTAGTAACTACGCATGCCCTTGTT	165
	R	FAM-TAACGCATTTGGAGCTAACCAAAACCTAAGG	
PV	F	Bio-GGGAATTGATTACTTCCATTATGTATTGGTGGT	185
	R	FAM-GGCCCAGTATGGTACAACCCAGAAGATAAA	

**Table 2:** Commercial products labeled as New Zealand mussel dishes and species identification results obtained by the dRAA–LFD assay.

Sample Code	Commercial Name (on Label)	Scientific Name	dRAA-LFD Result
P1	Baked New Zealand Mussels with Cheese	*P. canaliculus*	*P. viridis*
P2	Baked New Zealand Mussels with Cheese	*P. canaliculus*	*P. canaliculus*
P3	Spicy Kee Mao Spaghetti with New Zealand Mussels	*P. canaliculus*	*P. canaliculus*
P4	Thai Spicy New Zealand Mussel Salad	*P. canaliculus*	*P. canaliculus*
P5	Baked New Zealand Mussels with Cheese	*P. canaliculus*	*P. canaliculus*
P6	New Zealand Mussel Sushi	*P. canaliculus*	*P. viridis*
P7	New Zealand Mussel Steak	*P. canaliculus*	*P. canaliculus*
P8	Baked New Zealand Mussels with Cheese	*P. canaliculus*	*P. canaliculus*
P9	Pineapple Red Curry with New Zealand Mussels	*P. canaliculus*	*P. canaliculus*
P10	Salt-Grilled New Zealand Mussels	*P. canaliculus*	*P. canaliculus*
P11	Korean-Style Stir-Fried Noodles with New Zealand Mussels	*P. canaliculus*	*P. canaliculus*
P12	Spicy Mixed Seafood Soup	*P. canaliculus*	*P. canaliculus*
P13	New Zealand Mussel Omelette	*P. canaliculus*	*P. canaliculus*
P14	Stir-Fried New Zealand Mussels with Chili Paste	*P. canaliculus*	*P. canaliculus*
P15	Blanched New Zealand Mussels with Herbs	*P. canaliculus*	*P. canaliculus*

## Data Availability

The data presented in this study are available upon request from the corresponding author.
